# Skipping a Beat: Heartbeat-Evoked Potentials Reflect Predictions during Interoceptive-Exteroceptive Integration

**DOI:** 10.1093/texcom/tgaa060

**Published:** 2020-09-01

**Authors:** Leah Banellis, Damian Cruse

**Affiliations:** School of Psychology and Centre for Human Brain Health, University of Birmingham, Edgbaston B15 2TT, UK; School of Psychology and Centre for Human Brain Health, University of Birmingham, Edgbaston B15 2TT, UK

**Keywords:** attention, expectation, interoception, predictive coding, precision

## Abstract

Several theories propose that emotions and self-awareness arise from the integration of internal and external signals and their respective precision-weighted expectations. Supporting these mechanisms, research indicates that the brain uses temporal cues from cardiac signals to predict auditory stimuli and that these predictions and their prediction errors can be observed in the scalp heartbeat-evoked potential (HEP). We investigated the effect of precision modulations on these cross-modal predictive mechanisms, via attention and interoceptive ability. We presented auditory sequences at short (perceived synchronous) or long (perceived asynchronous) cardio-audio delays, with half of the trials including an omission. Participants attended to the cardio-audio synchronicity of the tones (internal attention) or the auditory stimuli alone (external attention). Comparing HEPs during omissions allowed for the observation of pure predictive signals, without contaminating auditory input. We observed an early effect of cardio-audio delay, reflecting a difference in heartbeat-driven expectations. We also observed a larger positivity to the omissions of sounds perceived as synchronous than to the omissions of sounds perceived as asynchronous when attending internally only, consistent with the role of attentional precision for enhancing predictions. These results provide support for attentionally modulated cross-modal predictive coding and suggest a potential tool for investigating its role in emotion and self-awareness.

## Introduction

The Bayesian brain hypothesis states that the brain is a probabilistic machine, with hierarchical neuronal representations underlying cognition, perception, and behavior (Friston 2009). The predictive coding framework posits that, in the comparison between top-down predictions from high-level brain regions and incoming low-level sensory input, any difference between the two signals is propagated up the hierarchy as a prediction error, thus allowing for iterative updating of the higher level representations (Rao and Ballard 1999). Successful matching of predictions with incoming stimuli, and thus successful minimization of prediction error, results in ‘correct’ perception, cognition, and action (Friston 2010). Minimization of prediction error is accomplished either by updating predictive models to accommodate unexpected signals (i.e., perceptual inference) or by performing actions (such as motor or autonomic responses) to better match predictions (i.e., active inference) (Friston 2010; Adams et al. 2013), consistent with an embodied view of cognition (Allen and Friston 2018).

As with the perception of external stimuli (exteroception), perception of internal stimuli (interoception) is also considered to be supported by hierarchical prediction error minimization mechanisms (Seth et al. 2012; Seth 2013; Barrett and Simmons 2015). Broadly, interoception is the perception of visceral bodily sensations such as heartbeat contractions, the expansion of lungs, or feelings of the body’s internal state such as hunger or nausea (Sherrington 1906; Cameron 2001). The Embodied Predictive Interoceptive Coding model describes an interoceptive cortical network comprising of viscerosensory neural afferents which arrive at the brainstem and thalamus via the dorsal root ganglion and vagus nerve, outputting to the hypothalamus, amygdala, anterior cingulate cortex, and the insula, with its highest regions residing in the posterior ventral medial prefrontal cortex and the orbitofrontal cortex (Critchley and Harrison 2013; Damasio and Carvalho 2013; Barrett and Simmons 2015; Quadt et al. 2018). This network is thought to be involved in numerous high-level cognitive processes such as emotional processing, bodily self-consciousness, visual awareness, self-recognition, attention, and time perception (Craig 2009; Critchley and Harrison 2013; Tsakiris and Critchley 2016; Quadt et al. 2018; Azzalini et al. 2019). Indeed, as part of a prediction error minimization framework, Seth et al. (2012) and Seth (2013) have proposed that embodied selfhood and emotional experience are the outcome of successful suppression of interoceptive prediction errors through active inference (Seth and Friston 2016). Additionally, dysfunctional interoceptive predictive mechanisms have been proposed to account for a variety of psychological disorders such as anxiety, depression, autism, dissociative disorders, and psychotic illnesses (Seth et al. 2012; Quattrocki and Friston 2014; Haker et al. 2016; Seth and Friston 2016), thus increasing scientific interest in characterizing these mechanisms.

One potential method of investigating the neural basis of interoceptive predictive mechanisms is by analyzing heartbeat-evoked potentials (HEPs) (Schandry et al. 1986; Pollatos and Schandry 2004). HEPs are averaged electrophysiological signals time-locked to heartbeats and are thought to reflect neuronal processing of cardiac afferents, encompassing interoceptive prediction error of each individual heartbeat (Ainley et al. 2016; Petzschner et al. 2019). In a recent study on interoceptive predictions, Pfeiffer and De Lucia (2017) presented healthy participants with a sequence of tones that were either synchronous or asynchronous with their own heartbeat. Crucially, the occasional tone was unexpectedly omitted from these sequences. Evoked responses to expected sounds that did not happen—that is, omission responses—are an elegant way of observing pure prediction signals without the contamination of auditory potentials (Wacongne et al. 2011; Chennu et al. 2016). Consequently, Pfeiffer and De Lucia (2017) reported a larger HEP during omission periods in cardiac synchrony, relative to cardiac asynchrony, consistent with a predictive account in which the brain uses the interoceptive (cardiac) signals to predict upcoming exteroceptive signals (sounds).

Predictions and their errors are also influenced by their precision—formally, the inverse of the variance, or the uncertainty in the signal. Within the prediction error minimization framework, attention is described as a means to optimize the relative precision weight of predictions and prediction error signals, via synaptic gain control (Friston 2009). For example, attending to a specific sensory signal is thought to enhance the precision of the predictions related to that signal, subsequently influencing associated prediction errors (Hohwy 2012). Consistent with the characterization of the HEP as a neural correlate of precision-weighted interoceptive prediction error, many studies have reported attentional modulation of the amplitude of the HEP—for example, during tasks involving attending to heartbeat sensations relative to external stimuli (Schandry et al. 1986; Montoya et al. 1993; Yuan et al. 2007; García-Cordero et al. 2017; Villena-González et al. 2017; Petzschner et al. 2019).

The relative weight of precision in perceptual inference is also influenced by individual differences in relative uncertainty (Lawson et al. 2014; Seth and Friston 2016). For example, individuals who are accurate at identifying when sounds are synchronous with their heartbeat (i.e., performance on the heartbeat detection task) also exhibit higher HEP amplitudes relative to individuals who are poor heartbeat perceivers, just as in an attentive versus inattentive contrast (Schandry et al. 1986; Katkin et al. 1991; Pollatos and Schandry 2004; Pollatos et al. 2005). Indeed, Ainley et al. (2016) have previously characterized these individual differences in interoceptive ability as individual differences in relative precision of prediction errors. However, caution should be taken when interpreting differences across interoceptive ability groups, as multiple heartbeat detection paradigms exist, which assess distinct processes and may not measure interoceptive ability validly (Brener and Ring 2016; Ring and Brener 2018; Corneille et al. 2020). In addition, Garfinkel et al. (2015) suggested three distinct and dissociable dimensions of interoceptive ability: interoceptive sensibility, accuracy, and awareness, with each dimension potentially influencing predictive mechanisms differently.

Consequently, a combined study of attention to interoceptive signals and individual differences in interoceptive ability allows us to directly test this predictive framework within the domain of evoked potentials. Specifically, here, we report the effect of attention and interoceptive ability on interoceptive predictions reflected in the electrical potentials evoked by omissions within a heartbeat detection task. As omission-evoked responses reflect top-down predictions from higher cortical regions, our approach allows us to measure the influence of attentional precision on interoceptive prediction and error signals, without contaminating bottom-up input (Wacongne et al. 2011; Chennu et al. 2016). Consistent with the characterizations of the precision-weighting nature of both within-subject and between-subject variations in attention (Feldman and Friston 2010; Hohwy 2012; Chennu et al. 2016), we hypothesized that HEPs during auditory omission periods would be 1) larger when sounds are perceived as synchronous with the heartbeat; 2) larger when the heartbeat is attended; and 3) larger for those individuals with high interoceptive ability. At the source level, we anticipated increased anterior insula activation when sounds are perceived as synchronous, supporting the role of the insula as a hub for interoceptive and exteroceptive integration (Gray et al. 2007; Salomon et al. 2016). Furthermore, we hypothesized increased activation in the insula, cingulate cortex, and somatosensory cortex (postcentral gyrus) when directing attention internally, than externally, and in individuals with high interoceptive perception, than poor interoceptive perceivers, as previously observed in fMRI studies (Critchley et al. 2004; García-Cordero et al. 2017).

## Materials and Methods

Unless otherwise stated, all methods, analyses and hypotheses were preregistered at https://osf.io/nr8my/.

### Participants

We recruited 39 participants from the University of Birmingham via advertisement on posters or the online SONA Research Participation Scheme. Our inclusion criteria were: right-handed 18–35-year-olds, with no reported cardiovascular or neurological disorders. We compensated participants with course credit. The Psychology Research Ethics Board of the University of Birmingham granted ethical approval for this study and written informed consent was completed by all participants. The data of 5 participants were excluded because of poor data quality resulting in more than a third of the trials of interest rejected. Subsequent analyses were completed on a final sample of 34 participants (Median age = 20 years, Range = 18–28 years). We chose this sample size in advance as it provides 80% power to detect a medium effect size (0.5) in our within-subjects interaction between attention and cardio-audio delay (alpha = 0.05; GPower, Faul et al. 2007).

### Stimuli and Procedure

The experiment consisted of four blocks of 56 trials (224 trials total), with each trial consisting of 7–10 auditory tones (1000 Hz, 100-ms duration, 44 100 sampling rate) presented via external speakers, with breaks given between each block. The onset of each tone was triggered by the online detection of the participants R-peak from electrocardiography (ECG) recordings using Lab Streaming Layer and a custom MATLAB script (Kothe et al. 2018). The script analyzed in real time the raw ECG signal by computing the variance over the preceding 33-ms window and determining if the signal exceeded an individually adjusted threshold, at which point a tone was triggered to occur after either an average time of 287-ms (perceived synchronous) or 587-ms (perceived asynchronous) delay (Brener and Kluvitse 1988; Wiens and Palmer 2001). Due to computational variability in online detection of R-peaks, R->Sound intervals had a standard deviation of 30 ms for both the perceived synchronous and asynchronous trials. In half of the trials, the third from last tone was omitted, resulting in an R-peak without an auditory stimulus. A fixation cross was present during tone presentation.

**Figure 1:**
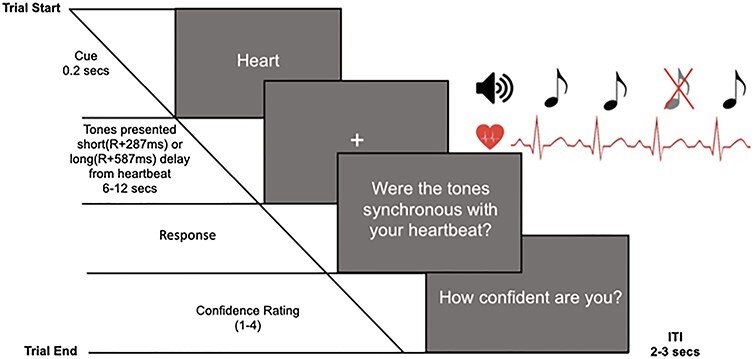
Experimental design of the integrated heartbeat detection (internal attention) and omission detection task (external attention), displaying an internal attention trial.

A cue at the start of each trial (200 ms) directed participants’ attention to focus internally (‘Heart’) or externally (‘Tone’). During the internal task, participants focused on their heartbeat sensations (without taking their pulse) and determined whether the tones presented were synchronous or not with their heartbeat. During the external task, participants were told to ignore their heartbeat sensations and direct attention toward the sounds alone. The external task was to determine whether there was a missing sound during that trial. Participants responded to the internal task (‘Were The Tones Synchronous With Your Heart?’) or external task (‘Was There a Missing Tone?’) question by pressing ‘y’ for yes or ‘n’ for no on the keyboard, and rated their confidence in their decision from 1 to 4 (1 = total guess, 2 = somewhat confident, 3 = Fairly Confident, 4 = Complete Confidence). The intertrial interval was between 2 and 3 s, chosen from a uniform distribution on each trial (see Fig. 1). The order of the experimental conditions was randomized to ensure no more than 3 of the same condition on consecutive trials. Finally, participants completed the short Porges Body Perception Questionnaire (BPQ), including a body awareness and autonomic reactivity subscale (Porges 1993).

### Indices

Interoceptive accuracy was calculated by comparing the normalized proportion of hits (responding ‘yes’ to a short cardio-audio delay ‘R + 287 ms’ trial) with the normalized proportion of false alarms (responding ‘yes’ to a long cardio-audio delay ‘R + 587 ms’ trial) (i.e., internal task *d*-prime [*d′*]) (Macmillan and Creelman 1990). Additionally, we calculated exteroceptive accuracy by comparing the normalized proportion of hits (responding ‘yes’ to a trial including an omission) with the normalized proportion of false alarms (responding ‘yes’ to a trial without an omission) (external task *d′*). The proportion of hits and false alarms were normalized using the inverse of the standard normal cumulative distribution.

As in previous studies (Garfinkel et al. 2015; Ewing et al. 2017), we quantified sensibility to a variety of internal bodily sensations with the score on the awareness subsection of the Porges BPQ (Porges 1993) and defined sensibility to heartbeat sensations as the median confidence rating during internal trials (Garfinkel et al. 2015; Forkmann et al. 2016; Ewing et al. 2017).

Interoceptive awareness was calculated using type 2 signal detection theory analysis comparing observed type 2 sensitivity (meta-*d′*) with expected type 2 sensitivity (*d′*) (Maniscalco and Lau 2012). Meta-*d′* is the *d′* expected to generate the observed type 2 hit rates and type 2 false alarm rates and was estimated using maximum likelihood estimation (Maniscalco and Lau 2014). This determined the extent to which confidence ratings predicted heartbeat detection accuracy, and thus interoceptive awareness. Groups were separated into high/low interoceptive accuracy, sensibility, and awareness with median splits.

### E‌EG/ECG Acquisition

EEG was recorded throughout the experiment using a gel-based 128-channel Biosemi ActiveTwo system, acquired at 512 Hz, referenced to the Common Mode Sense electrode located ~2 cm to the left of CPz. Two additional electrodes recorded data from the mastoids, and ECG was measured using two electrodes placed on either side of the chest, also sampled at 512 Hz.

### EEG/ECG Pre-Processing

First, we filtered the continuous EEG data in two steps (i.e., high-pass then low-pass) between 0.5 and 40 Hz using the finite impulse response filter implemented in EEGLAB (function: pop_eegfiltnew). We filtered ECG between 0.5 and 150 Hz (Kligfield et al. 2007). Next, we segmented the filtered EEG signals into epochs from −300 to 800 ms relative to the R-peak of the ECG recording during the omission period, re-referenced to the average of the mastoids. We detected the R-peaks using a custom MATLAB script and subsequently checked the accuracy of R-peak detection via visual inspection. When necessary, we manually corrected the estimated R-peaks to ensure accurate R-peak detection. To account for online heartbeat detection errors (i.e., missed or multiple sounds per R-peak), we rejected blocks with R–R intervals >1.5 or <0.4 s from both behavioral and EEG analyses. The subsequent artifact rejection proceeded in the following steps based on a combination of methods described by Nolan et al. (2010) and Mognon et al. (2011).

First, bad channels were identified and removed from the data. We consider a channel to be bad if its absolute z-score across channels exceeds 3 on any of the following metrics: 1) variance of the EEG signal across all time-points; 2) mean of the correlations between the channel in question and all other channels; and 3) the Hurst exponent of the EEG signal (estimated with the discrete second-order derivative from the MATLAB function *wfbmesti*). After removal of bad channels, we identified and removed trials containing non-stationary artifacts. Specifically, we considered a trial to be bad if its absolute *z*-score across trials exceeds 3 on any of the following metrics: 1) the mean across channels of the voltage range within the trial; 2) the mean across channels of the variance of the voltages within the trial; and 3) the mean across channels of the difference between the mean voltage at that channel in the trial in question and the mean voltage at that channel across all trials. After removal of these individual trials, we conducted an additional check for bad channels and removed them, by interrogating the average of the channels across all trials (i.e., the evoked response potential (ERP), averaged across all conditions). Specifically, we considered a channel to be bad in this step if its absolute *z*-score across channels exceeds 3 on any of the following metrics: 1) the variance of voltages across time within the ERP; 2) the median gradient of the signal across time within the ERP; and 3) the range of voltages across time within the ERP.

To remove stationary artifacts, such as blinks and eye-movements, the pruned EEG data are subjected to independent component analysis with the *runica* function of EEGLAB. The MATLAB toolbox ADJUST subsequently identified which components reflect artifacts on the basis of their exhibiting the stereotypical spatio-temporal patterns associated with blinks, eye-movements, and data discontinuities, and the contribution of these artifact components is then subtracted from the data (Mognon et al. 2011). Next, we interpolated the data of any previously removed channels via the spherical interpolation method of EEGLAB, and re-referenced the data to the average of the whole head.

We included an additional preprocessing step beyond those planned in our preregistration to control for differences in the cardiac field artifact (CFA) at our different delay conditions (Nakamura and Shibasaki 1987). Specifically, we calculated single-subject average HEPs during rest periods, following the same preprocessing pipeline as the experimental HEPs. In a similar approach to that used in previous research (Van Elk et al. 2014), we then subtracted the average resting HEP from individual experimental trials, locked to each heartbeat. This conservative method eliminates remaining artifacts due to additional heartbeats within the same trial.

Before proceeding to group-level analyses, single-subject CFA-corrected averages for HEP analysis are finalized in the following way. First, a robust average was generated for each condition separately, using the default parameters of SPM12. Robust averaging iteratively down-weights outlier values by time-point to improve the estimation of the mean across trials. As recommended by SPM12, the resulting HEP was low-pass filtered below 20 Hz (again, with EEGLAB’s pop_neweegfilt). In a deviation from our preregistration, but following discussions with peer reviewers and investigation of similar decisions in previous studies of HEPs (Park et al. 2014; Babo-Rebelo et al. 2016, 2019; Azzalini et al. 2019), we chose not to apply any baseline correction to our data as cardiac activity is cyclical by nature and may therefore insert artifactual effects in post-R data.

### HEP Analysis

HEPs during the omission period were compared with the cluster mass method of the open-source MATLAB toolbox FieldTrip (Oostenveld et al. 2011: fieldtrip-20181023). This procedure involves an initial parametric step followed by a nonparametric control of multiple-comparisons (Maris and Oostenveld 2007). Specifically, we conducted either two-tailed dependent samples *t*-tests (for comparison 1) or a combination of two-tailed independent and dependent samples *t*-tests (for comparison 2) at each spatio-temporal data-point within the time window. Spatiotemporally adjacent *t*-values with *P*-values <0.05 are then clustered based on their proximity, with the requirement that a cluster must span more than one time-point and at least 4 neighboring electrodes, with an electrode’s neighborhood containing all electrodes within a distance of 0.15 within the Fieldtrip layout coordinates (median number of neighbours = 11, range 2–16). Finally, we summed the *t*-values at each spatio-temporal point within each cluster. Next, we estimated the probability under the null hypothesis of observing cluster sum Ts more extreme than those in the experimental data—that is, the *P*-value of each cluster. Specifically, fieldtrip randomly shuffles the trial labels between conditions, performs the above spatio-temporal clustering procedure, and retains the largest cluster sum T. Consequently, the *P*-value of each cluster observed in the data is the proportion of the largest clusters observed across 1000 such randomizations that contain larger cluster sum T’s.

Our preregistered analyses were to be conducted on the ERP data from 100 to 600 ms relative to the R-peak. However, it subsequently became evident that this approach is confounded by the lag difference in tone presentation across conditions. Consequently, here, we report one set of analyses on ERP data from 0 to 229 ms post-R (i.e., the first percentile of the short delay R-sound intervals, thus before 99% of anticipated tones) and a second set of analyses from 0 to 213 ms relative to the onset of the omitted sound (i.e., from 287 to 500 ms post-R for the short delay condition and 587–800 ms post-R for the long-delay condition).

### Comparisons

Using the above method, HEPs were compared across cardio-audio delay and attention conditions to assess the main effects, and the interaction was calculated as the difference between short-delay and long-delay trials between attention groups (i.e., a double-subtraction; comparison 1). If an interaction was observed, pairwise separate analyses were completed to consider simple effects. Similar comparisons were completed across attention and interoceptive individual difference conditions (interoceptive awareness, accuracy, and sensibility) (comparison 2).

### CFA Control Analyses

We performed control analyses on the ECG data, to determine if differences in cardiac activity contributed toward the HEP results. Therefore, equivalent analyses to that performed on the HEPs were completed on the ECG data. Subsequently, single-subject robust averages of the ECG activity were computed for each condition and were analyzed using the cluster mass method, as described above. The same comparisons were completed as to those which showed a significant HEP effect (i.e., ECG was compared across cardio-audio delay conditions 0–229 ms post-R, and the attention and delay interaction was assessed 0–213 ms relative to the omission).

### HEP Control Analyses

As our analyses involved comparing HEPs at different latencies relative to the R-peak, it is possible that artifactual effects could be inserted due to the relative position of the underlying HEP, rather than due to differing cognitive processes. To test this concern, we performed the same analyses on HEPs recorded prior to the onset of any sounds in the trial—that is, before any task-related processing could become evident. Specifically, single-subject robust averages of presound HEP activity relative to the first R-peak after the cue were computed for each condition and analyzed using the cluster mass method, and using the same comparisons as those which showed significant HEP effects (cardio-audio delay conditions were compared 0–229 ms post-R, and the attention and delay interaction was assessed using the same window as the omission-locked analysis [i.e., from 287 to 500 ms post-R for the short delay condition and 587–800 ms post-R for the long delay condition]).

To further control for residual HEP differences and reinforce our main effect of delay, we analyzed the difference between delay conditions before the first and fourth sound. We chose the fourth sound as the omission could occur from the fifth sound onward. Therefore, robust averages were computed relative to the R-peak for the first and fourth sound. We averaged presound HEP activity belonging to the electrodes and time-window of the significant preomission positive and negative clusters separately, for each participant. Subsequently, a two-way ANOVA analyzed the interaction of cardio-audio delay (short and long delay) and sound number (first and fourth sound) and post hoc *t*-tests analyzed the effect of cardio-audio delay separately for the first and fourth sound.

### Source Reconstruction

Since our initial preregistration, we discovered that our planned source analysis pipeline performed poorly at localizing basic sensory responses in a separate study in our lab. Consequently, we concluded that those preregistered methods were inappropriate for this study. Therefore, here, we report a more rudimentary but validated source reconstruction method, using statistical parametric mapping (SPM12) (Henson et al. 2009; López et al. 2014).

Our source estimation approach was completed for each time-window separately in which we observed a significant sensor level effect: 27–230 ms post-R for the main effect of delay, 95–138 ms relative to the omission for the attention and delay interaction (i.e., 382–425 ms post-R for the short delay condition and 682–725 ms post-R for the long delay condition), and 102–138 ms relative to the omission for the follow-up simple effects analysis (i.e., difference between cardio-audio delay conditions for internal and external trials separately: 389–425 ms post-R for the short delay condition and 689–725 ms for the long-delay condition). For each time-window, within SPM12, we applied a hanning taper to downweight the signal at the beginning and end of the window in the condition-wise grand averages, and filtered the data between 1 and 48 Hz. Cortical sources of each sensor-level HEP were reconstructed using the default anatomical template in SPM. Electrode positions were co-registered to the template using the fiducials of the nasion, left peri-auricular and right peri-auricular points. We calculated the forward model using the Boundary Element Method. The inverse model was generated based on an empirical Bayesian approach. Specifically, we applied the greedy search fitting algorithm, which optimizes the multiple sparse priors approach when localizing the sensor-level evoked responses. Finally, we contrasted the condition-wise source estimates (i.e., generated difference source volumes). The estimated source results were projected onto a canonical inflated brain surface for visualization, using the open source MNI2FS toolbox (Price 2020: https://github.com/dprice80/mni2fs).

## Results

### Behavioral Data

Participants’ interoceptive accuracy scores (internal *d′*) were significantly greater than zero (*M* = 0.218, SD = 0.347; *t*(33) = 3.665, *P* < 0.001, BF_10_ = 36.52). This indicates that performance on the heartbeat discrimination task was above chance and therefore confirms our interpretation of the R + 287 ms cardio-audio delay as perceived synchronous and R + 587 ms as perceived asynchronous. Additionally, performance on the external task (external *d′*) was significantly greater than zero (*M* = 3.091, SD = 1.105; *t*(33) = 3.665, *P* < 0.001, BF_10_ = 2.273e+14), indicating that participants were attentive during both tasks.

There was no significant difference in exteroceptive performance between the short-delay (*M* = 2.943, SD = 1.056) and long-delay trials (*M* = 2.990, SD = 0.938) (*t*(33) = −0.470, *P* = 0.642). A Bayesian equivalent analysis indicated the data were 5× (1/0.204) more likely to occur under a model with no effect of cardio-audio delay (BF_10_ = 0.204), demonstrating that external task performance is likely to be independent from heartbeat perception. Also, there was no significant correlation between internal and external task accuracy, further suggesting that performance on the internal and external task was independent (*r*(32) = 0.299, *P* = 0.085, BF_10_ = 0.883).

### Heart-Evoked Potentials

#### Cardio-Audio Expectation

**Figure 2:**
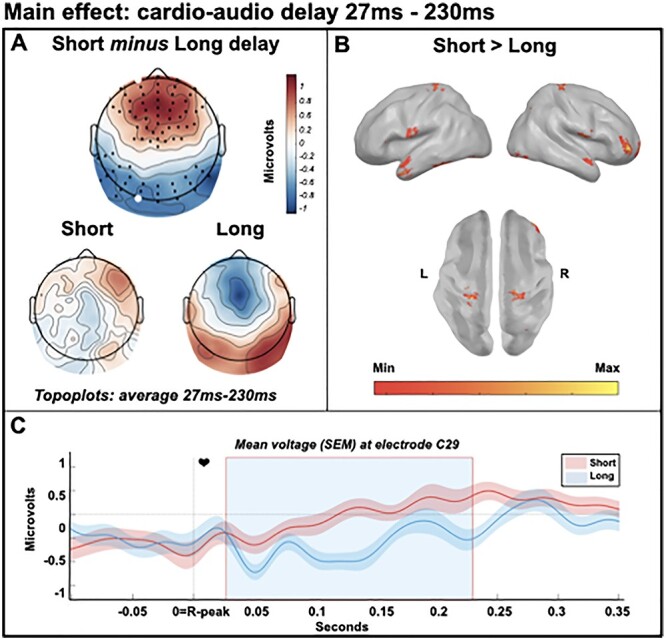
Main effect of cardio-audio delay from 27 to 230 ms, reflecting differences in cardio-audio expectation. (*A*) Scalp distribution of the average significant difference across delay conditions 27--230 ms, with electrodes contributing to the dipolar clusters marked. (*B*) Estimated sources of the main effect in bilateral primary somatosensory cortex, bilateral primary motor cortex, bilateral supramarginal gyrus, right anterior prefrontal cortex, and bilateral middle temporal cortex. (*C*) Average HEP across participants at electrode C29, light blue shaded region represents the time of the significant positive effect.

We observed a significant early dipolar main effect of cardio-audio delay (positive cluster *P* = 0.001, and negative cluster *P* = 0.005), perhaps reflecting a difference in expectation induced by the heartbeat. Estimated generators of this effect include bilateral primary somatosensory cortex, bilateral primary motor cortex, bilateral supramarginal gryus, right anterior prefrontal cortex, and bilateral middle temporal cortex. The positive cluster extended from 27 to 230 ms and the negative cluster 93 to 169 ms post R-peak, with the cardio-audio delay conditions reflected in qualitatively different topographic distributions, supporting our hypothesis of the role of cardiac signals to predict auditory stimuli. We observed no significant main effect of attention on preomission responses (smallest cluster *P* = 0.062) (see Fig. 2).

#### Unfulfilled Expectation

**Figure 3:**
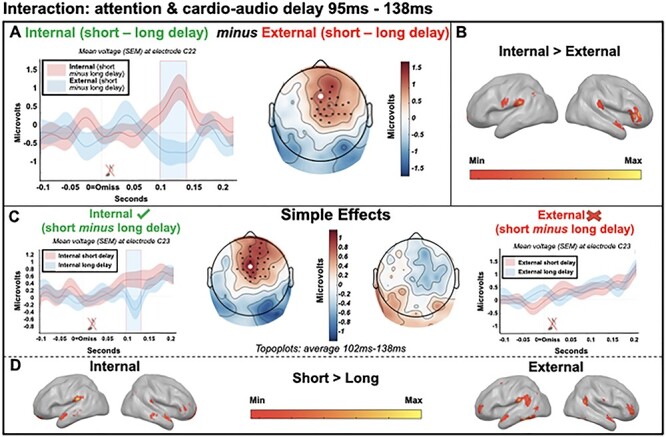
Interaction between attention and cardio-audio delay from 95 to 138 ms, relative to the omitted sound. (*A*—left) Average omission-evoked response across participants at electrode C22, light blue shaded region represents the time of the significant effect. (*A*—right) Scalp distribution of the average significant interaction (attention × delay) 95–138 ms, with electrodes contributing to the cluster marked. (*B*) Estimated sources of the interaction in the right inferior frontal gyrus, bilateral supramarginal gyrus, and right middle temporal cortex. (*C*) Analysis of the simple effects showing qualitatively different topographical distributions across attention conditions (102–138 ms) and a significant effect of delay in the internal attention condition only. (*D*—left) Estimated sources of internal simple effects analysis in bilateral supramarginal gyrus, right inferior frontal gyrus and bilateral orbitalfrontal cortex, bilateral anterior prefrontal cortex, and bilateral middle and superior temporal cortex. (*D*—right) Estimated sources of external simple effects analysis in left premotor cortex, bilateral angular gyrus, left supramarginal gyrus, bilateral anterior prefrontal cortex, left fusiform gyrus, and bilateral temporopolar area.

The cluster-based permutation test indicated a significant, though weak, interaction between cardio-audio delay and attention (cluster *P* = 0.017) with estimated sources in right inferior frontal gyrus, bilateral supramarginal gyrus, and right middle temporal cortex, supporting our hypothesis of attentional modulation of predictive mechanisms. The cluster in the observed data extended from 95 to 138 ms postomission. Follow-up simple effects tests indicated a larger positivity within this cluster for short-delay omissions relative to long-delay omissions during internal attention only (cluster extended 102--138 ms, *P* = 0.007), while there were no clusters formed when contrasting the cardio-audio delay conditions when externally attending. This supports our hypothesis of larger HEPs during omission periods within short-delay (perceived synchronous) than long-delay (perceived asynchronous) trials. Source analyses estimated internal simple effects in bilateral supramarginal gyrus, right inferior frontal gyrus, bilateral orbitalfrontal cortex, bilateral anterior prefrontal cortex, and bilateral middle and superior temporal cortex, while external simple effects were estimated in bilateral angular gyrus, left supramarginal gyrus, left premotor cortex, bilateral anterior prefrontal cortex, left fusiform gyrus, and bilateral temporopolar area (see Fig. 3).

### Control ECG Comparisons

We observed no difference clusters when comparing ECG responses between cardio-audio delay conditions, 0–229 ms post-R. Similarly, no clusters were found when analyzing the interaction between attention and cardio-audio delay on ECG responses, 0–213 ms relative to the omitted sound. Therefore, we conclude that it is unlikely that ECG activity contributed toward the HEP differences observed.

### Interoceptive Ability

Cluster-based permutation tests indicated no significant interaction of high and low interoceptive awareness (smallest *P* = 0.388), accuracy (smallest *P* = 0.231), or sensibility (both median confidence rating and the awareness subsection of the BPQ; smallest *P* = 0.138) with attention, during short-delay trials.

**Figure 4:**
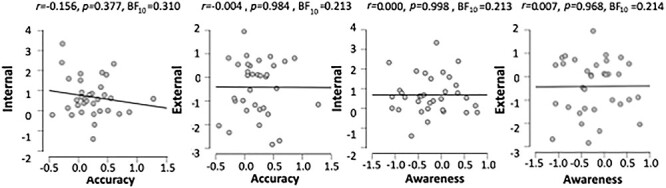
Correlations of interoceptive accuracy and interoceptive awareness with the mean difference in voltage across cardio-audio delay conditions during the significant interaction window for internal and external trials.

We also completed exploratory correlations of interoceptive ability with the amplitude of each participant’s delay effect during the interaction time window (95–138 ms relative to the omission). These analyses reveal no significant correlation between the delay effect and interoceptive accuracy (*r*(32) = −0.004, *P* = 0.984, BF_10_ = 0.213) or interoceptive awareness (*r*(32) = 0.007, *P* = 0.968, BF_10_ = 0.214) during external attention, or the delay effect and interoceptive accuracy (*r*(32) = −0.156, *P* = 0.377, BF_10_ = 0.310) or awareness (*r*(32) = −0.000, *P* = 0.998, BF_10_ = 0.213) during internal attention (see Fig. 4). Additionally, no significant correlations were found with interoceptive sensibility (both the awareness subsection score and the autonomic reactivity subsection score of the BPQ) (smallest *P* = 0.300) for both internal and external trials. Additionally, there was no significant equivalent correlations during the main effect of delay time window (27–230 ms relative to the R-peak) (smallest *P* = 0.162). This is inconsistent with our hypothesis of interoceptive ability modulating predictive responses.

### HEP Control Analyses

#### Cardio-Audio Expectation

We would predict that a true expectation effect (as we interpret our preomission effect to be) would be evident in the R->Sound periods of all sounds in that trial, perhaps increasing in magnitude over the trial as more evidence accumulates about whether the trial is short or long delay. Therefore, we analyzed the main effect of delay before the fourth sound and compared this with the delay effect before the first sound of each trial, using the significant electrodes and time-window of the positive and negative preomission clusters. This analysis indicated a significant interaction between trial position and delay (positive cluster: *F*(1,33) = 5.447, *P* = 0.026; negative cluster: *F*(1,33) = 6.022, *P* = 0.020), indicating a greater difference between the delay conditions before the fourth sound (positive difference = 0.460; negative difference = 0.641) than before the first sound (positive difference = 0.243, negative difference = 0.211), consistent with our view that the preomission effect reflects an expectation that has built-up across the trial.

However, follow-up *t*-tests indicated a significant differences between the delay conditions for both the first sound (positive cluster: *t*(33) = 3.598, *P* = 0.001; negative cluster: *t*(33) = −2.469, *P* = 0.019) and the fourth sound (positive cluster: *t*(33) = 8.526, *P* < 0.001; negative cluster: *t*(33) = −6.530, *P* < 0.001). Topographically, the delay effect before the fourth sound is very similar to that we observe before the omission, whereas the delay effect before the first sound has a qualitatively distinct topography, indicative of not entirely overlapping cognitive processes or neural generators (see Supplementary Material, Supplementary Fig. 1).

#### Unfulfilled Expectation

As the omission-locked analyses involved analyzing HEPs at different moments (R + 287 ms for the short-delay condition and R + 587 ms for the long-delay condition), it is possible that our effect could be due to comparing early and late HEP components, irrespective of cardio-audio integration. To control for this, we analyzed the attention and delay interaction using the same time-windows (R + 287 and R + 587 ms) relative to the first R-peak post cue (before any sounds) and found no significant interaction (*P* = 0.609). Therefore, we interpret this control analysis as evidence that the omission-locked attention and delay interaction is not a result of comparing HEPs at different moments post-R.

### Interbeat Intervals

Because previous research found differences in the interbeat intervals (IBIs) following omissions and deviant stimuli, we additionally investigated this as an exploratory analysis (Pfeiffer and De Lucia 2017; Raimondo et al. 2017). IBIs were significantly longer during internal attention (M = 834.356 ms, SD = 108.000 ms) than during external attention (M = 813.442 ms, SD = 102.074 ms; *F*(1,33) = 69.475, *P* < 0.001, partial *n*^2^ = 0.678, BF_inclusion_ = 3.002e+15). However, there was neither significant IBI difference between the cardio-audio delay conditions (*F*(1,33) = 2.342, *P =* 0.135, partial *n*^2^ = 0.066, BF_inclusion_ = 0.484) nor was there a significant interaction between attention and cardio-audio delay (*F*(1,33) = 3.223, *P* = 0.082, partial *n*^2^ = 0.089, BF_inclusion_ = 0.479).

**Figure 5:**
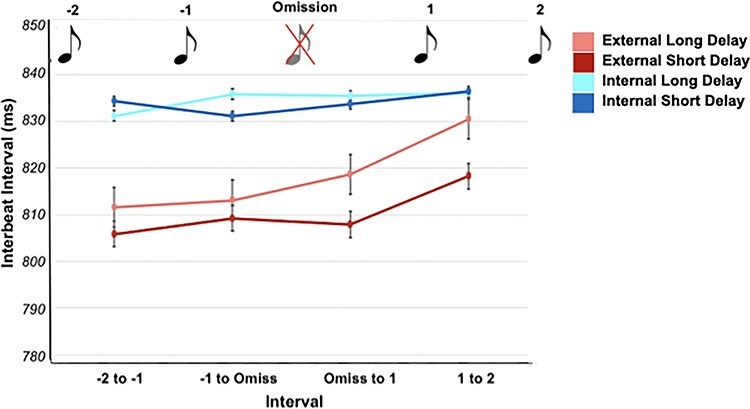
IBIs in relation to the omission, with error bars reflecting standard error.

Additionally, we calculated the IBI’s relative to the omission, revealing an IBI increase postomission when attending externally. A three-way ANOVA analyzed the IBIs post-omission (IBI ‘omission to 1’ and IBI ‘1 to 2’) across cardio-audio delay and attention conditions (see Fig. 5). This revealed a main effect of IBI (*F*(1,33) = 17.320, *P* < 0.001, partial *n*^2^ = 0.344, BF_inclusion_ = 7.739), a main effect of attention (*F*(1,33) = 25.391, *P* < 0.001, partial *n*^2^ = 0.435, BF_inclusion_ = 1.481e+10), a main effect of delay (*F*(1,33) = 4.605, *P* = 0.039, partial *n*^2^ = 0.122, BF_inclusion_ = 6.579), a significant delay and attention interaction (*F*(1,33) = 5.062, *P* = 0.031, partial *n*^2^ = 0.133, BF_inclusion_ = 9.945), and a significant attention and IBI interaction (*F*(1,33) = 13.717, *P* < 0.001, partial *n*^2^ = 0.294, BF_inclusion_ = 2.819). The delay and IBI interaction was not significant (*F*(1,33) = 0.339, *P* = 0.565, partial *n*^2^ = 0.010, BF_inclusion_ = 0.471), and the delay, attention, and IBI interaction was not significant (*F*(1,33) = 0.007, *P* = 0.932, partial *n*^2^ < 0.001, BF_inclusion_ = 0.329) (see Fig. 5).

Post hoc *t*-tests revealed that the first IBI after the omission (IBI omission to 1) was significantly faster (short delay: *M* = 818.674, SD = 106.089; long delay: *M* = 807.908, SD = 96.939) than the following IBI (IBI 1 to 2) (short delay: *M* = 830.529, SD = 107.646; long delay: *M* = 818.255, SD = 100.182) for external attention trials during both cardio-audio short-delay stimulation (*t*(33) = −4.820, *P* < 0.001, BF_10_ = 726.098) and long-delay stimulation (*t*(33) = −3.535, *P* = 0.001, BF_10_ = 26.644). There was no significant difference between the postomission IBIs during internal attention trials ([short delay: *t*(33) = −0.981, *P* = 0.334, BF_10_ = 0.286]; [long delay: *W* = 234, *P* = 0.285, BF_10_ = 0.187]). This appears to reflect a cardiac deceleration when the omission was a target (i.e., during external attention, see Fig. 5).

Post hoc *t*-tests also revealed a significant IBI difference between the cardio-audio delay trials during external attention (IBI omiss to 1: *t*(33) = 2.640, *P* = 0.013, BF_10_ = 3.559; IBI 1 to 2: *W* = 442, *P* = 0.012, BF_10_ = 1.764), whereas there was no significant IBI difference between cardio-audio delay trials during internal attention (IBI omiss to 1: *t*(33) = −.474, *P* = 0.639, BF_10_ = 0.204; IBI 1 to 2: *t*(33) = 0.082, *P* = 0.935, BF_10_ = 0.184).

### Heart Rate Variability

We analyzed the standard deviation of the IBI’s (SDRR) as a measure of heart rate variability (HRV). A two-way ANOVA on the SDRR values revealed that the IBI’s was significantly more variable when internally attending (short delay: *M* = 77.18, SD = 20.81; long delay: *M* = 78.64, SD = 25.96) than when externally attending (short delay: M = 73.07, SD = 21.94, long delay: M = 76.32 SD = 26.65) (*F*(1,33) = 5.481, *P* = 0.025, *n*^2^ = 0.142). However, a Bayesian equivalent analysis revealed only weak evidence of an effect of attention on HRV (BF_inclusion_ = 1.361). While there was no significant interaction between attention and delay (*F*(1,33) = 0.546, *P* = 0.465, *n*^2^ = 0.016, BF_inclusion_ = 0.295), the HRV difference between delay conditions was larger when externally attending (difference = 3.25) than when internally attending (difference = 1.46). As these effects are in the opposite direction to those reported in the HEPs, we conclude that the HRV task differences are unrelated to our HEP effects. Finally, there was no significant main effect of cardio-audio delay (*F*(1,33) = 2.135, *P* = 0.153, *n*^2^ = 0.061, BF_inclusion_ = 0.497).

## Discussion

Several theories propose that emotion and embodied self-awareness arise from the integration of internal and external signals and their respective precision-weighted expectations (Seth et al. 2012; Seth 2013; Barrett and Simmons 2015; Seth and Friston 2016). Here, we investigated these mechanisms of integrated interoceptive and exteroceptive expectations by comparing HEPs during heartbeat-predicted omissions, thus allowing a measure of pure prediction signals without the contamination of bottom-up auditory inputs (Wacongne et al. 2011; Chennu et al. 2016).

First, we observed a preomission HEP difference when comparing cardio-audio delay trials, reflected in qualitatively different topographical distributions (see Fig. 2*A*). Consistent with the hypothesis that interoceptive signals guide expectations of exteroceptive stimuli, this result indicates that different expectations of upcoming sounds are induced by different cardio-audio delays and that these differential expectations are supported by not entirely overlapping regions of cortex. Pfeiffer and De Lucia (2017) reported a similar HEP difference during omission periods when comparing cardio-audio synchronous stimulation with asynchronous stimulation, supporting the integration of cardiac signals to predict auditory stimuli. However, because the sounds in that study (and therefore omission responses) were time-locked to the R-peak during synchronous stimulation but shuffled relative to the R-peak in the asynchronous condition, the auditory omission response is confounded in that contrast. We control for this in our study by comparing trials with sounds at fixed cardio-audio intervals, ensuring the auditory omission response is time-locked to the heartbeat in both delay conditions. This allows for the comparison of preomission periods, and later omission-locked responses, which subsequently excludes the auditory omission response as a confound. Nevertheless, our HEP differences across perceived synchrony are consistent with that reported by Pfeiffer and De Lucia (2017). Similarly, in another study consistent with heartbeat-driven auditory predictions, Van Elk et al. (2014) observed a weak auditory N1 suppression to heartbeat-locked sounds, in comparison to cardio-audio asynchronous sounds, although not statistically significant in that study (*P* = 0.07).

As further evidence that the preomission effect of delay reflects differential expectations, we also observed that this effect increases in magnitude across the trial, perhaps as evidence accumulates regarding the short/long-delay nature of the trial. Nevertheless, we also observe a significant effect of delay in the period before the first sound of the trials—that is, before any expectation could be formed—with a qualitatively distinct topography indicative of distinct processes and generators to the effect we observe later in the trial and preomission. While this result does not affect our interpretation of the preomission expectation effect, as the effects across the trial are clearly electrophysiologically distinct, it highlights the possibility for significant HEP effects to be generated by factors not related to the task. Consequently, considerable control analyses are required in studies of HEPs to moderate cognitive interpretations (discussed further below).

We also observe an interaction between attention and cardio-audio delay when comparing omission-locked evoked responses. This is present as a larger positivity to short-delay omissions than long-delay omissions, when attending internally only. This supports our hypothesis of stronger unfulfilled expectations of a tone in trials presenting sounds at a short perceived synchronous delay than at a longer perceived asynchronous delay. These results are additionally consistent with the role of top-down attentionally mediated mechanisms in generating expectations of upcoming stimuli. This is supported by modeling evidence, highlighting that omissions are generated by top-down driving inputs, which are attentionally modulated via strengthened downward connections (Chennu et al. 2016). Source estimates of the attentionally mediated omission-locked response revealed the orbitofrontal cortex and inferior frontal gyrus during internal attention only, while the anterior prefrontal cortex and supramarginal gyrus were consistently implicated in all cardio-audio delay contrasts (i.e., during the R-locked main effect of delay [Fig. 2*B*] and the omission-locked internal and external simples effects [Fig. 3*D*]), suggesting that the prediction of a sound in relation to the heartbeat may originate from these areas. This is broadly consistent with previous cardiac attention research which highlight the prefrontal cortex, although usually the inferior or middle frontal gyrus (Critchley et al. 2004; Pollatos et al. 2007; Wiebking et al. 2010; Zaki et al. 2012; Simmons et al. 2013; Kuehn et al. 2016; Schulz 2016). Additionally, the supramarginal gyrus has previously been implicated during a variety of interoceptive attention/awareness tasks and is thought to be related to the multisensory integration of information from the body and the environment (Reichenbach et al. 2011; Nejad et al. 2015; Salvato et al. 2020).

Previous research indicated that attention enhances mismatch and omission responses, further supporting the role of attention at modulating predictive mechanisms (Raij et al. 1997; Garrido et al. 2009; Chennu et al. 2013; Chennu et al. 2016). Despite this, Pfeiffer and De Lucia (2017) reported a heart-beat driven prediction error effect in a group of participants who were naive to the presence of omissions, contrary to our results of absent heartbeat-driven effects when not attending to the heartbeat. Nevertheless, our observation that attention did not modulate the magnitude of our preomission HEP effect but did modulate the amplitude of the omission-locked ERP effect is consistent with a view that the expectation of an upcoming sound can be instantiated without direct attention but that attention differentially enhances the precision of those expectations so that their violations (i.e., omissions) lead to ERP effects that are modulated by attention (Kok et al. 2012).

The modulating nature of attention on HEPs is consistent with previous research and with the interpretation of the HEP as a marker of precision-weighted prediction error of each individual heartbeat (Schandry et al. 1986; Montoya et al. 1993; Yuan et al. 2007; García-Cordero et al. 2017; Villena-González et al. 2017; Petzschner et al. 2019). Attention is proposed to modulate predictive mechanisms by enhancing the precision of attended prediction errors, relative to the precision of their priors (Hohwy 2012; Ainley et al. 2016; Petzschner et al. 2019). Subsequently, attending to internal signals could enhance the precision of interoceptive prediction errors, resulting in their propagation up the predictive hierarchy to update models for more accurate future predictions regarding each heartbeat. The enhanced cardiac predictions would in turn allow for more precise auditory predictions of heartbeat-locked sounds, such as those presented in our task. Therefore, the enhanced predictions of each heartbeat due to internal attention allow for more precise priors regarding the timing of sounds relative to those heartbeats. The larger positivity to short-delay omissions may be because heartbeat-driven predictions of external stimuli are only stable/accurate across relatively short intervals from the heartbeat (i.e., ~287 ms). Similarly, Critchley et al. (2004) found a greater difference in fMRI activity between cardio-audio delay conditions when attending internally, than externally. This was reflected as an increase in the frontal operculum and insula, dorsal and medial parietal lobe, right dorsolateral prefrontal cortex, dorsal cingulate, and lateral temporal cortices during internal attention relative to external. This cortical network overlaps broadly with the source estimates of our interaction of attention with cardio-audio delay in the right inferior frontal gyrus, bilateral supramarginal gyrus, and middle temporal cortex.

As individual differences in the ability to perceive heartbeat sensations can also be framed as differences in precision, we expected interoceptive accuracy and awareness to similarly modulate interoceptive predictive mechanisms. However, we found no relationship between interoceptive ability and the HEP differences observed in our task. The lack of evidence for a relationship between our ERP effects and participants’ interoceptive abilities during internal attention is inconsistent with previous evidence that interoceptive accuracy modulated HEP responses (Schandry et al. 1986; Katkin et al. 1991; Pollatos and Schandry 2004; Pollatos et al. 2005). However, previous research used heartbeat counting tasks to assess interoceptive performance, rather than the heartbeat discrimination task used in our study, which likely confounds ability to estimate heart rate or time with the ability to sense individual heartbeats (Brener and Ring 2016; Ring and Brener 2018; Corneille et al. 2020). The lack of observed differences between interoceptive ability groups in our study could also be because of individual differences in the timing of heartbeat sensations, likely due to biological differences (Wiens and Palmer 2001). Therefore, some individuals may have performed poorly because they perceived both delay conditions as asynchronous (Brener et al. 1993; Brener and Ring 2016). This could be investigated in future research by previously determining each individual’s perceived synchronous delay (using the method of constant stimuli (Brener et al. 1993), for example) and subsequently individually adjusting the ‘perceived synchronous’ cardio-audio delay used for each individual (Brener and Kluvitse 1988; Mesas and Chica 2003). It is also possible that HEP differences related to interoceptive ability occur at later latencies than we could measure in our design. For example, ERPs related to metacognition are thought to occur at late latencies (between 550 and 1900 ms), which would overlap with ERPs evoked by successive auditory stimuli in our design (Sommer et al. 1995; Skavhaug et al. 2010; Tsalas et al. 2018). Furthermore, interoceptive metacognitive awareness has previously been associated with long-range connectivity patterns (global activity), rather than HEP local activity differences (Canales-Johnson et al. 2015). Future investigation of these connectivity markers in our data may reveal further relationships.

A potential limitation of our task design is that the internal and external tasks differ in their difficulty. However, we argue that if our observed HEP differences are the result of a task difficulty confound, then we would expect that these effects would also correlate with interoceptive performance, which they do not. A further potential limitation is that the omission is task-relevant in the external task only, perhaps reflected in the post-omission cardiac deceleration during external trials. However, we do not observe any HEP differences as a result of cardio-audio delay during external attention, which would not be expected if task relevance of the omission was an influence on the predictive effects reflected in the HEP. Future research could use an alternative external task of increased difficulty with equal omission task-relevance, such as determining the synchronicity of sounds with a faint flashing visual stimulus, excluding task-related differences as a potential confound. Additionally, the tasks may have differed in the temporal scale used to make each decision, with the external task perhaps requiring a longer time-period of integration to correctly identify omitted sounds relative to the time-period of integration required to judge cardio-audio synchronicity. However, the precise periods of temporal integration and their electrophysiological effects across tasks are unclear without further future quantification.

Previous research has stressed the importance of controlling for ECG artifacts when comparing HEP responses (Kern et al. 2013; Van Elk et al. 2014). We corrected for ECG artifacts using a similar method to that used by Van Elk et al. (2014), by subtracting the average HEP response during rest periods for each participant. Our correction was potentially more conservative as it was time locked to each heartbeat within individual trials. Considering the ECG correction applied, the lack of heart rate or HRV differences in the direction of the ERP effects, and the lack of statistical difference between ECG responses across conditions of interest, we conclude that our observed HEP differences are unlikely to be due to differences in ECG activity, but rather reflect predictive mechanisms of the integration of internal and external stimuli. Another potential confound is a consequence of comparing different moments of the HEP (i.e., R + 287 vs. R + 587 ms). Thus, one could argue that the observed omission-locked interaction may simply reflect different components of the underlying HEP. However, our control analysis indicated no evidence of a significant interaction when applying the same analyses to HEP data before presentation of any sounds, thus strengthening our cognitive interpretation of this effect.

Our results support the mechanisms underlying interoceptive predictive coding accounts that suggest that embodied selfhood and emotional experience are a result of integrated self-related predictions from multiple modalities (including interoceptive, exteroceptive, and proprioceptive signals) (Seth et al. 2012; Seth 2013; Barrett and Simmons 2015; Seth and Friston 2016). This is supported by studies which demonstrated the contribution of integrative interoceptive signals with visual cues to enhance body ownership and self-recognition (Aspell et al. 2013; Suzuki et al. 2013; Sel et al. 2017; Heydrich et al. 2018). Additionally, interoceptive and exteroceptive integration has been suggested to explain the generation of a first-person perspective, describing how our unified conscious experience of the external world is integrated with the experience of the self, with particular focus on interoception as a binding agent (Azzalini et al. 2019). These viewpoints, therefore, demonstrate the potential function of the integrated interoceptive and exteroceptive mechanisms observed in our study.

Investigating HEP differences across cardio-audio delay conditions may be a useful clinical tool for assessing dysfunctional interoceptive-exteroceptive predictive mechanisms. As mentioned, the experience of emotion or selfhood is proposed to be the result of the integration of interoceptive predictive mechanisms with exteroception and proprioception (Seth and Friston 2016). Therefore, measuring pure predictive signals during omissions, which reflect interoceptive and exteroceptive integration, may be useful for diagnosing dissociative disorders, schizophrenia, or anxiety (Paulus and Stein 2010; Synofzik et al. 2010; Seth et al. 2012; Seth 2013; Petzschner et al. 2017). Additionally, if interoceptive and exteroceptive integrative mechanisms contribute toward a unified conscious first-person perspective, then observing preserved mechanisms could be useful for diagnosing awareness in patients with disorders of consciousness (Azzalini et al. 2019). This would be advantageous because current methods of assessing awareness focus almost exclusively on responses to external stimuli, whereas assessing interoceptive and exteroceptive integration could provide a method of assessing both external perceptual and internal self-related aspects of awareness.

In conclusion, our results demonstrate that interoceptive signals can guide the expectations of exteroceptive stimuli and that attentional-precision modulates integrative cross-modal predictive mechanisms. Nevertheless, we found no evidence that the HEPs were related to subjective experience of heartbeat sensations suggesting low validity of our two-alternative-forced-choice method of assessing interoceptive awareness, or that there exists a more subtle interaction of HEPs and subjective experience. The integrative interoceptive and exteroceptive predictive mechanisms described here provide a useful tool for assessing embodied and interoceptive predictive coding accounts of cognition and clinical disorders.

## Funding

The Medical Research Council IMPACT Doctoral Training Programme at the University of Birmingham (scholarship to L.B.); Medical Research Council New Investigator Research (grant MR/P013228/1; to Principal investigator D.C.).

## Data Availability

All data and scripts are available at https://osf.io/v9khb/.

## Supplementary Material

Supplementary_Material_tgaa060
