# Supplementary material for: Skipping a Beat: Heartbeat-Evoked Potentials Reflect Predictions during Interoceptive-Exteroceptive Integration
Source: Cereb Cortex Commun. 2020 Sep 1;1(1):tgaa060. doi: 10.1093/texcom/tgaa060 (PMC8153056; doi:10.1093/texcom/tgaa060)
Supplement: Supplementary_Material_tgaa060 [file supplementary_material_tgaa060.docx]

**Supplementary Material**

*Supplementary Figure 1: Topoplots of the average activity 27ms-230ms relative to the R-peak before the first sound and before the forth sound for each cardio-audio delay condition.*

**
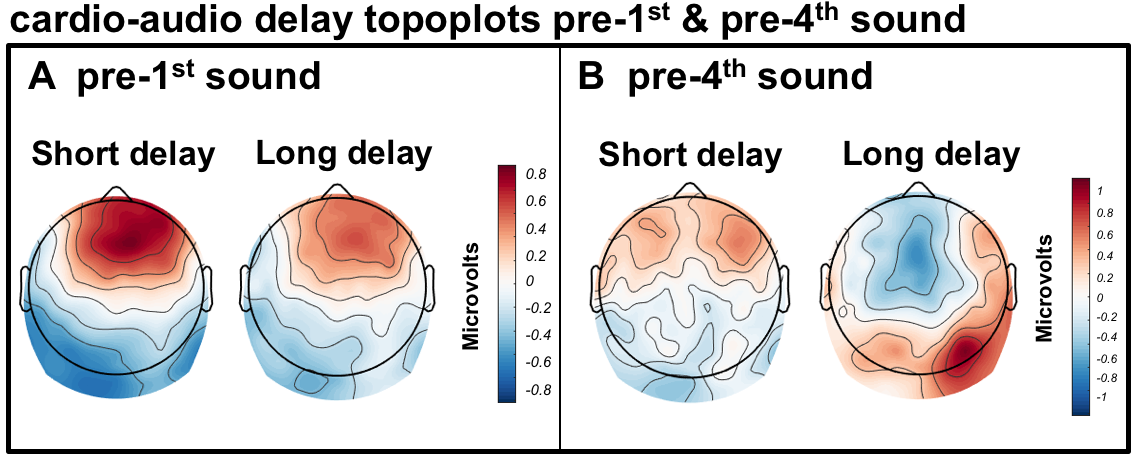
**
